# A Review of Optical NDT Technologies

**DOI:** 10.3390/s110807773

**Published:** 2011-08-08

**Authors:** Yong-Kai Zhu, Gui-Yun Tian, Rong-Sheng Lu, Hong Zhang

**Affiliations:** 1 School of Automation Engineering, Nanjing University of Aeronautics and Astronautics, Nanjing 210016, China; E-Mail: zhuyongkai@nuaa.edu.cn; 2 School of Electrical, Electronic and Computer Engineering, Newcastle University, Newcastle NE1 7RU, UK; E-Mail: hong.zhang1@ncl.ac.uk; 3 School of Instrument Science and Opto-Electronics Engineering, Hefei University of Technology, Hefei 230009, China; E-Mail: rslu@hfut.edu.cn

**Keywords:** optical non-destructive testing (NDT), speckle, endoscopic, infrared thermography, terahertz (THz) technology

## Abstract

Optical non-destructive testing (NDT) has gained more and more attention in recent years, mainly because of its non-destructive imaging characteristics with high precision and sensitivity. This paper provides a review of the main optical NDT technologies, including fibre optics, electronic speckle, infrared thermography, endoscopic and terahertz technology. Among them, fibre optics features easy integration and embedding, electronic speckle focuses on whole-field high precision detection, infrared thermography has unique advantages for tests of combined materials, endoscopic technology provides images of the internal surface of the object directly, and terahertz technology opens a new direction of internal NDT because of its excellent penetration capability to most of non-metallic materials. Typical engineering applications of these technologies are illustrated, with a brief introduction of the history and discussion of recent progress.

## 1. Introduction

NDT (non-destructive testing) techniques, used over 30 years, are analysis techniques used in science and industry to evaluate the properties of a material, component or system without causing damage to the samples [1]. The term non-destructive evaluation (NDE) is also commonly used to describe this technology. Because NDT refers to the detection of faults without affecting the operation of the equipment, it has been used in a variety of fields [2,3]. Currently, widely used NDT methods include ultrasonic, eddy current, microwave and acoustic emission, *etc*. However, these methods have limitations on the type of objects that can be inspected or the type of defect to be detected. Recent developments in optical NDT technology give higher detection accuracy and sensitivity, plus ease of signal multiplexing and resistance to electromagnetic interference. Main types of optical NDT are surface measurements, such as infrared thermal imaging, endoscopic and speckle imaging for component surfaces and subsequent image analysis to determine the presence of a defect. Optical fibre sensing is used for dynamic parameter measurements.

This paper outlines various optical NDT technologies including small-scale optical fibre NDT, speckle NDT based on laser interferometry mechanism, infrared thermography NDT, endoscopic NDT, and THz NDT, which has been a very hot topic in recent years, and their applications in different fields. Section 2 focuses on the theory and application of different NDT technologies, and Section 3 briefly summarizes development of these technologies.

## 2. Optical Methods and Applications

In this section the principles of different optical NDT techniques, including optical fibre, speckle, infrared thermography, endoscopic, THz in NDT, are discussed. Typical measurement systems are demonstrated. The development and application of these optical NDT techniques are described.

### 2.1. Optical Fibre NDT

Optical fibre NDT features the use of optical fibres to collect and sense the signal light from the object under test (OUT). The optical fibre is resistant to electromagnetic interference and corrosion, which means that in can be used in extreme environments. The optical fibre is small and lightweight and could be either attached to the surface of the tested structure or be embedded inside, having little impact on the structure itself. Fibre optic sensors can be designed to measure or sense just about anything. A few examples include liquid level, chemical, pressure, electric field, vibration strain and temperature. There are many types of sensor designs, including Bragg, IFPI (Intrinsic Fabry Perot Interferometer) and EFPI (Extrinsic Fabry Perot Interferometer) [4]. While there are many types of optical sensors, the focus of this section is on the fibre Bragg grating sensor.

**Figure 1.**
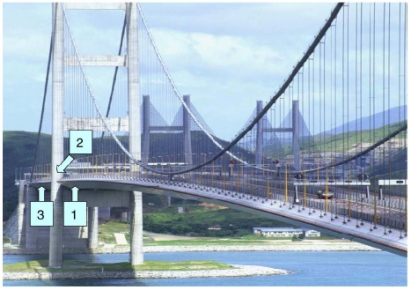
The FBG sensors installed on the Tsing Ma bridge at (**1**) hanger cable, (**2**) rocker bearing, (**3**) truss girders of section Chainage 23488. Reproduced with permission from [9], copyright 2006, Elsevier.

**Figure 2.**
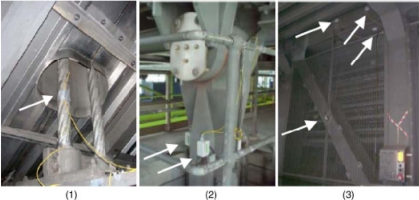
The detail view of FBG sensors installed on (**1**) hanger cable, (**2**) rocker bearing, (**3**) truss girders of section Chainage 23488. Reproduced with permission from [9], copyright 2006, Elsevier.

When the physical quantities to be measured are strain, temperature, pressure, *etc*., the optical fibre itself can be the sensor. In these cases, the physical quantities to be measured can cause a change of the intensity, phase, polarization, wavelength, or transit time of the light in the fibre system, and NDT is implemented through monotoring these optical parameters.Light intensity monitoring is the simplest and most frequently used technology in practice. The most successful application of optical fibre NDT is the field of civil engineering, where it has been used or over 20 years [4–7]. Usually the fibre bundles are buried in the concrete structure; the strain and temperature of these structures would modulate the light intensity, wavelength, phase, polarization, *etc*. passing through the fibre and through the analysis of these optical signals properties of the structure such as overload and corrosion damage can be measured for condition monitoring and NDT. The University of Vermont research group of Fuhr and Huston has studied the optical fibre NDT for highways, railways, bridges, dams and buildings [8]. At present, many bridges use optical fibre sensors, such as Tsing Ma Bridge in Hong Kong, as shown in Figures 1 and 2 [9].

Similarly, there is much research on optical fibre NDT for composite materials; where the fibre is embedded into the composite structure during manufacture or attached to the surface to measure the strain and structural damage. By using a longer Fibre Bragg grating (FBG) we can achieve a large multi-point strain measurement and damage detection, especially useful in monitoring large structures such as aircraft, composite pipes, ships and wind turbine blades. Optical fibre NDT for composite measurement has on average 1 με resolution; Tay *et al.* employed a serpentine design of an optical fibre sensor to further improve the resolution of these optical sensors [10]. Murukeshan *et al.* applied a fiber polarimetric sensor for smart structure damage detection [11]. Thursby *et al.* used lamb waves to detect holes with optical fibre polarization sensors embedded within the structure or attached to the surface [12].

**Figure 3.**
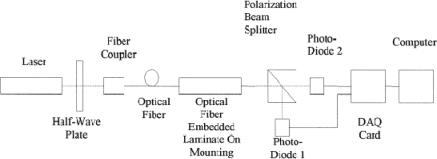
A structure of optical fibre polarization sensor detecting system. Reproduced with permission from [11], copyright 2000, Elsevier.

Figure 3 shows the optical setup for the polarization-based fibre optic sensor. It is formed by polarizing the light from a light source (polarized He-Ne laser) via a half-wave plate. The half wave plate here is used to rotate the polarization state of the plane polarized He-Ne light. That could be a length of polarization-preserving fibre. The fibre here serves as the sensing medium. Under external perturbation such as stress or strain, the phase difference between two polarization states is changed, and so the output polarization state is changed accordingly. Hence, by using PC analyzing the output polarization state at the exit end of the fibre, the external perturbation can be detected.

Optical fibre sensing is usually used as a non-destructive testing method at multiple locations for long time monitoring, for single or multiple parameters like temperature and strain. To monitor the status of a large object or system, like a natural gas pipeline, multiple discrete optical fiber sensors are placed at different locations of the object or system, forming a distributed sensor network. Real-time parameters like temperature and strain are collected by the sensors and transmitted to a data processing center through an optical transmission network. This method uses the optical fibre as the sensing medium to monitor more than one physical parameters, as well as the transmission medium to combine the distributed sensor network. It has advantages of real-time monitoring, large data processing capability, high-integration, and excellent environment tolerance. Therefore, distributed fibre sensing technology is a hot research area today, and the multi-sensor network addressing (or multiplexing) and location (or demodulation) methods are important research fields in NDT.

### 2.2. Speckle NDT

Laser speckle inteferometry makes use of the intensity distribution pattern formed from space interference, generated by illumination of coherent light onto rough object surfaces. Laser speckle interferometry is an effective NDT technology with advantages of non-contact, the illumination over the surface of the object need not be uniform, high sensitivity and detection rate. It has been widely used in many industry areas. Laser speckle interferometry can be used in inspection of metal, ceramic, glass, rubber and composite materials. The surface stress can be also measured for highly accurate measurements of deformation, which means it has great application potential in aerospace, automotive, marine and high-tech materials manufacture.

**Figure 4.**
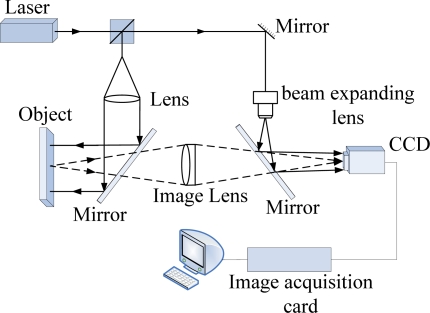
Out-of-plane ESPI system.

In the laser speckle interferometer field, the two most important technologies are ESPI (Electronic Speckle Pattern Interferometry) and ESPSI (Electronic Speckle Pattern Shearing Interferometry). What should also be noted is the technology to combine speckle pattern interferometry together with digital image correlation (DIC), which has been the subject of intense research interest these years [13]. Electronic Speckle Pattern-Interferometry (ESPI) combines double ray interference technology with digital recording devices, and can be classified as in-plane ESPI and out-of-plane ESPI. Take the out-of-plane ESPI as the example, with the operating principle shown in Figure 4. The object light reflected by object surfaces superimposes with the reference light which directly emits into CCD target, forming double ray interference patterns. ESPI is a whole-field optical technique widely used for measuring displacement components, shape and slope contours of surfaces, *etc*. This non-contact and highly sensitive technique has developed into a powerful online inspection tool for non-destructive evaluation. The salient feature of ESPI is its capability to display the correlation fringes in real time on a TV monitor without the need of photographic processing or optical filtering. In conventional ESPI use the displacement of the laser speckle to study the displacement of object surface. This method used charge-coupled device (CCD) camera to record the object speckle before and after force is applied; and then electronically process and compare the measurements; similar to interference fringes, the final results will be display in the TV screen.

**Figure 5.**
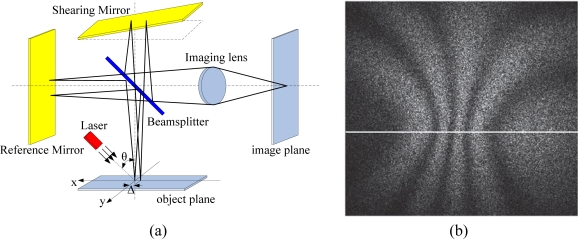
(**a**) Out-of-plane Electronic Shearography; (**b**) A shearography correlation fringle pattern. Reproduced with permission from [18], copyright 2010, IOP.

In order to improve the anti-seismic performance of ESPI, in 1985 Hung employed staggered technology and proposed the concept of ESPI dislocation (Electronic Shearography), also known as electronic dispersion spot shearing interferometer [14]. This technique has been widely used in NDT for in-line quality control and tracking. Electronic speckle pattern shearing interferometry (ESPSI), also known as digital shearography, is a full-field non-destructive, optical technique used to measure approximately the field of displacement derivatives. It can be divided into out-of-plane displacement gradient measurement, in-plane displacement gradient measurement, and multi-component measurement [15]. The operating principle of the out-of-plane displacement gradient measurement system is shown in Figure 5. A laser illuminates the object after passing through a beam expander mirror, and the diffuse reflected light splits into two beams at the splitter. The two beams are reflected by the reflecting mirrors and then get focused by the images lens onto the image plane of the imaging system. As shown in Figure 5, Michaelson interferometer can be used for image shearography. Tilting of one of the shear reflecting mirror will cause the two speckles in the image plane to shear each other, and the two sheared speckles superimpose to form the speckle interference patters, which is recorded by the CCD camera. Deformation of the object will also cause the speckle pattern to change. Shearography uses CCD camera to digitize the speckle patterns before and after the object deformation, which is then processed by computer, as shown by Figure 5(b) (the white horizontal line is added by author). The difference of the two speckle patterns would reproduce the strap pattern in the shear direction which corresponds to the derivative of the displacement, and therefore the object deformation is measured [16–18].

This method directly measures the surface deformation gradient, with precise phase shifting and image analysis, it has advantages of real-time testing with post-processing of digital results. Moreover, it is not affected by the object rigid body dynamics and the results are reliable. Testing results can quickly show on the screen, and can represent the defect size by accurately quantify the bending strain. Yang and Hung have developed a range of practical speckle shearing interferometery instruments [17].

Shearography and ESPI systems are full-field optical techniques that can be used for out-of-plane contour and slope measurements. ESPI measures surface profiles directly, while shearography measures gradients of the slopes [18]. For those objects without planar shapes, contours and slopes are required for a complete surface strain analysis.

ESPI and ESPSI have been extensively applied for NDT. Gulker used the ESPI technique for on-site monitoring of buildings, due to the use of phase shifting, the sensitivity of ESPI is increased by two orders of magnitude [19], resulting in rapid development of the technology. After that ESPI technique was used for composite materials inspection, rough surface measurement and weld quality NDT [20]. Yang *et al.* developed the first three-dimensional speckle interferometer instrument for three-dimensional displacement and shape testing for measurement of complex surfaces [21]. Rao *et al.* successfully detected honeycomb plate defect under the thermal and vacuum loading, using a 632.8 nm laser and high-resolution (1,000 lines/mm) CCD for propellant tank defect location detection, with a detection range of up to 150 mm^2^ [22].

**Figure 6.**
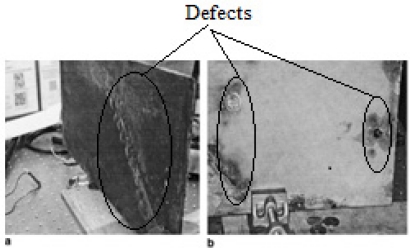
The testing sample for ESPI. Reproduced with permission from [23], copyright 2006, Elsevier.

Singh Raman *et al.* applied ESPI for detection of industrial paint stripping conditions on ships, applying an ESPI system to do high accuracy testing for 0.15 m × 0.2 m specimens, detection accuracy up to 0.4 μm, the a testing sample is shown in Figure 6 [23].

Lu employed electronic speckle pattern interferometer and laser technology on the carrier to perform quantitative measurement of the displacement field for diesel oil pumps, using high-power solid-pumped green laser (wavelength 532 nm) with a measurement range up to the 200 mm^2^ [24].

**Figure 7.**
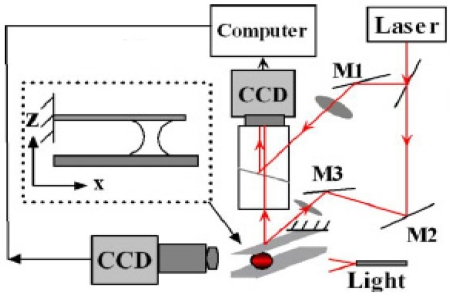
Experimental setup of micro-ESPI system. Reproduced with permission from [25], copyright 2006, American Institute of Physics.

Li and Peng constructed a micro-ESPI system to study the capillary adhesion of microcantilevers and substrate [25]. With the help of ESPI, they were able to measure the real-time mechanical response of the microbeam and the domain related to the adhered area, and thus to calculate the capillary force. The measured dynamic and equilibrium deformations could be compared directly with the theoretical results. They presented less than one micron deflections measured in couple of seconds using the micro-ESPI system. The experimental setup is shown in Figure 7.

Huang detected thermal deformation anomalies by shearography. Various samples with cracks and debonds (including aluminum plate, steel pipes, PVB pipes and FRP-reinforced concrete) are tested. Both qualitative and quantitative measurement can be processed. The results show that the technology has good practicability for NDT [26],

The above two technologies focus more on how to physically measure the speckle patterns. In recent years, a digital image processing method to combine the speckle pattern interferometry together with the Digital Image Correlation (DIC), has caused more and more interest. The digital image correlation (DIC) techniques was first conceived and developed by Yamaguch, Peter and Ranson *et al.* at the University of South Carolina in the early 1980s. The digital speckle image focused on studying one-dimensional field measurements. When there is small deformation of the object, the digital speckle image measures the light intensity before and after the deformation and uses the peak cross-correlation function to derive the displacement of the object. While DIC is a full-field image analysis method, based on grey value digital images, which can determine the contour and the displacements of an object under load in three dimensions. DIC is an extension of one-dimensional method to achieve higher accuracy. The DIC obtains the grey values of the object before and after deformation/displacement/strain, and then uses iteration method to calculate the correlation coefficient changes and find out the maximum correlation coefficient values to measure the corresponding deformation, displacement and strain [27–32]. It has been widely applied in many areas of science and engineering measurements.

**Figure 8.**
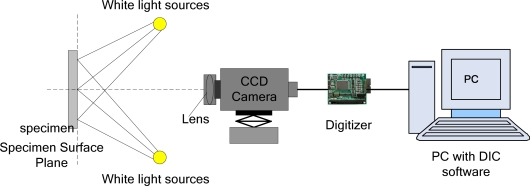
Schematic of 2-D DIC system.

**Figure 9.**
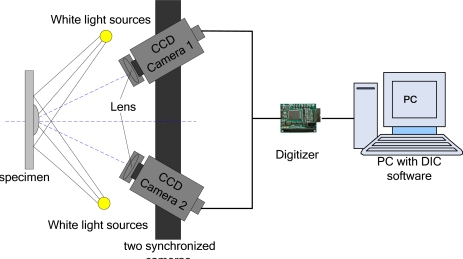
Schematic of a complete 3-D DIC system.

With the development of high-performance instrumentation (high-speed CCD, computer technology), image processing for large amounts of data can be achieved, DIC measurements have evolved from two-dimensional to three-dimensional. Luo and Chao first presented three-dimensional surface-displacement measurement from a stereo pair of CCD cameras in 1993 [33]. Currently, a typical two-dimensional structure of DIC indicate detection system shown in Figure 8, for a flat plane surface displacement measurement, only need one camera; three-dimensional structure of DIC signal detection system shown in Figure 9, it requires two or more cameras capture images. DIC technique can employ white light illumination, but it requires existence of speckle textures on the sample surface [34].

With the help of DIC technology people can measure mechanical properties of different materials [35–41], fatigue and breakup of different materials [42–47], even for high-temperature objects [48], among which the measurement of strain and flaw can provide rich information for NDT. Although digital image processing methods have been widely applied in many areas, to further improve the application in practice, people are pursuing less process time and high precision.

From the development of these digital speckle image measurement technologies and other correlated measurement technologies, the digital speckle measurement method has become a useful new technique in the field of modern photomechanics. Because of the use of CCD technology and computer vision technology, it is useful for non-contact and remote measurement, various types of materials in various types of environments; application of this method has developed from conventional materials to new materials, from room temperature to high temperature measurement and use at the macro-, micro- and nano-scales [49–52]. Digital speckle measurement techniques will play an important role in the field of materials mechanical properties measurement in the NDT of structures.

### 2.3. Infrared Thermography NDT

IR thermography is a technique for producing an image of the invisible to our eyes infrared light emitted by objects due to their thermal condition. Infrared thermography NDT is a new discipline, with the advantages of being fast, and providing non-contact, non-interaction, real-time measurements over a large detection area with a long range. Currently, infrared thermography NDT is widely used in aviation, aerospace, machinery, medical, petrochemical, power and other fields. United States, Russia, France, Canada and other countries have applied the infrared thermography technology widely in aircraft composite structures the internal defects and adhesive bonding quality testing, skin riveting quality testing. Both Airbus and Boeing have developed infrared aircraft components maintenance and detection standards, American Society for Testing and Materials (ASTM) has also developed infrared testing standards for the materials and components used in aircraft composites [53,54].

A typical type of thermography camera is a device that produces a live video picture using infrared radiation intensities. Infrared energy is just one part of the electromagnetic spectrum. All objects emit a certain amount of black body radiation as a function of their temperatures [55,56]. Higher sophisticated cameras can actually measure the temperature values of any object or surface in the image field-of-view and produce false colour images that make interpretation of thermal patterns easier. Infrared thermography is a process in which an infrared imaging system (an infrared camera) converts the spatial variations in infrared radiance from a surface into a two-dimensional image, in which variations in radiance are displayed as a range of colors or tones. As a general rule, objects in the image that are lighter in color are warmer, and darker objects are cooler. An image produced by an infrared camera is called a thermograph.

Infrared thermography is based on the temperature difference. There are two kinds of thermography: active and passive [57,58]. Active Thermography (AT) is defined as applying a stimulus to a target to cause the target to heat or cool in such a way as to allow characteristics of the target to be observed when viewed by thermal imagery. These observed characteristics may be flaws sought in Non Destructive Testing (NDT) or norms sought in quality control. Passive Thermography (PT) is defined as measuring the temperature differences between the target materials the surroundings under different ambient temperature conditions. Currently commonly used infrared thermography methods are active methods. Active thermography, according to different heating methods, can be divided into PT (Pulsed Thermography), PPT (Pulse-phase-Thermography) and MT (Modulated Thermography).In recent years, the development of thermography method has been an increasing interest in ULT (Ultrasonic Lock-in Thermography).

**Figure 10.**
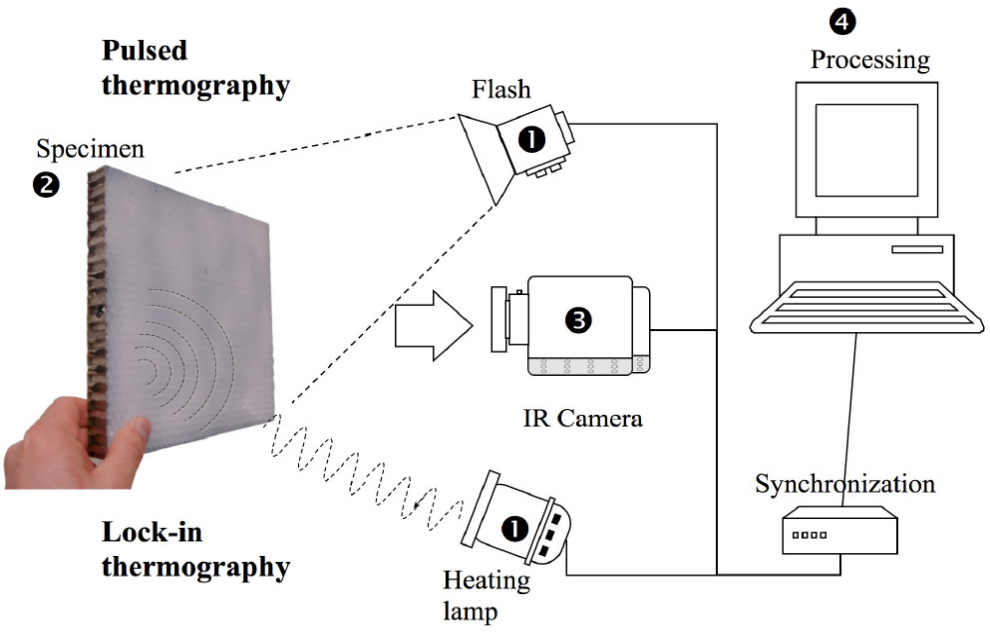
The experimental detection devices. Reproduced with permission from [58], copyright 2010, IOP.

**Figure 11.**
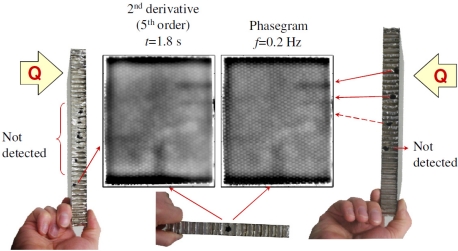
The surface thermal images of the sample. Reproduced with permission from [58], copyright 2010, IOP.

Sfarra *et al.* used infrared thermography to detect the cellular structure of honeycomb structures [58]. The sample is a 3-layer structure (thickness 18 mm), and the outer two layers are carbon fibre and the middle layer is aluminium. This method can detect a 4 mm diameter hole at 6 mm depth and a 6 mm diameter hole at 10 mm depth. Figure 10 illustrates two pulsed thermography (PT) and lock-in thermography (LT) configurations. Figure 11 shows two results obtained by IRT. Data was processed using two different processing techniques: thermographic signal reconstruction (TSR) and pulsed phase thermography (PPT).

**Figure 12.**
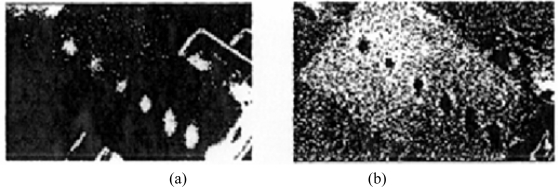
Minor collision defects in the carbon fibre composite plate thermal images: (**a**) Thermographic magnitude image; (**b**) Phase image. Reproduced with permission from [59], copyright 1998, Elsevier.

Rantala *et al.* [59] used thermography technology with modulated ultrasonic excitation simultaneously to heat the carbon fibre composite board sample; by analysing the phase of thermal wave, material information from combined heat and mechanical excitation is obtained. This method is characterized by stronger signal and deeper detection depth, and can detect smaller collision and lamination defects than individual methods. They tested a 0.125 mm thickness carbon fibre composite board [(±45/0/90) s] from eight different orientations. There are seven of these that have visual injury impact. Figure 12 shows the seven minor collision defects in the carbon fibre composite plate thermal images; in (a) and (b) by comparing the results of thermal wave phase, the size and temperature of the defects are obtained.

**Figure 13.**
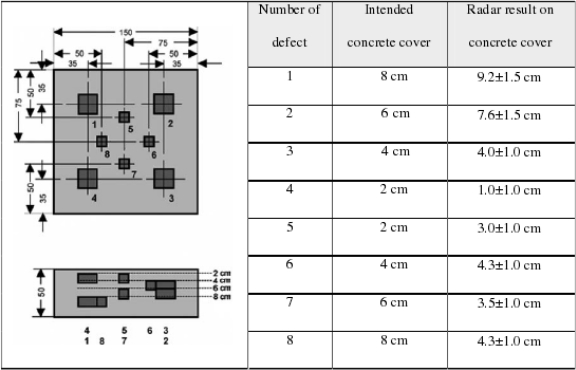
A sample with eight different sizes and depths holes. Reproduced with permission from [60], copyright 2006, Elsevier.

**Figure 14.**
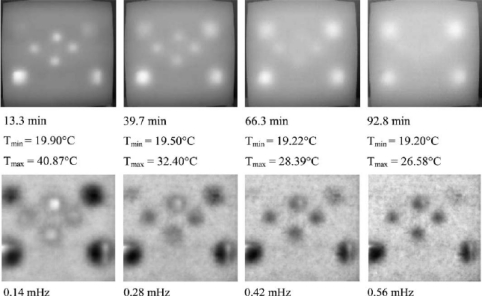
The thermographs of concrete samplea, top ones are thermograms, bottom ones are respective phase images. Reproduced with permission from [61], copyright 1999, Elsevier.

Maierhofer *et al.* [60] studied infrared thermography testing for voids and cellular hollows. Eight different sized holes were placed in a concrete sample, with different heating times at the top and bottom of the sample; producing different thermal infrared images. They also produced thermal conductivity curves for different density of concrete. This study suggests that this method is suitable for measuring defects in building materials near the surface (depth <10 cm); but for the deeper defects it may require 1.5 hours of heating time; for 2 cm depth flaws, it just need a few minutes of heating time. Figure 13 shows the concrete sample with eight different sizes and depths of holes. Figure 14 shows that by comparing the temperature and phase of the thermal waves, the defects in concrete can be found in the thermal images.

**Figure 15.**
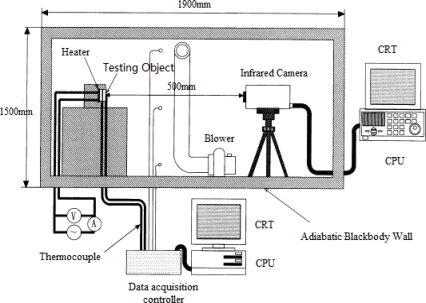
Japanese Heights State University thermography NDT systems. Reproduced with permission from [61], copyright 1999, Elsevier.

Inagaki *et al.* built an advanced thermography NDT system [61] (shown in Figure 15). The distance between the infrared camera and test sample *L* is 500 mm. The surface of measuring region is covered by a pseudo-blackbody surfaces with a black velvet texture that surrounds the boundary wall and the region is filled with glass wool insulation. To keep the inner surface temperature constant, the inner surface layer is also covered by the same pseudo-blackbody material to eliminate the multiple reflections between the surfaces. The air around is blown through the inhalation and natural cooling heat exchanger to maintain a uniform temperature. The air flow rate is small so that it does not affect the internal environment. The sample is tilted by 15° on the vertical axis and a ceramic heater placed behind the sample defect is used to heat the surface to the desired temperature.

Smith *et al.* [62] used thermography to inspect blades with a composite sandwich structure. They heated sandwich structure samples and analysed thermal response in the cooling stage, to identify defects and their characteristics.

PEC thermography involves the application of a high frequency (typically 50–500 kHz), high current, electromagnetic wave to the material under inspection for a short period (typically 20 ms−1 s) [63]. Where the induced eddy currents encounter a discontinuity, they are forced to divert, leading to areas of increased and decreased eddy current density. Areas where eddy current density is increased experience higher levels of Joule (Ohmic) heating, thus the defect can be identified from the IR image sequence, both during the heating period and during cooling.

**Figure 16.**
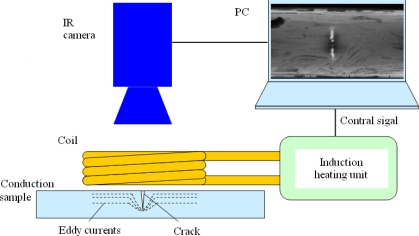
Basic configuration of pulsed eddy current thermography system. Reproduced with permission from [63], copyright 2010, Elsevier.

The configuration of a pulsed eddy current thermography system, shown in Figure 16 is very simple and consists of an induction heating system which induces eddy currents in the sample under inspection, the generated heat at the surface of the material is captured by an IR camera, with data displayed and stored on a PC [63].

Recent development of infrared NDT features the integration of infrared and other testing methods, the change of signal processing method from amplitude information to phase information, and more and more stress on field testing oriented systems.

### 2.4. Endoscopic NDT

Endoscopic NDT uses optical measurements through a hole to see the internal structural properties of a sealed object; these optical images are then evaluated to analyze possible defects of the object. The biggest advantage of this technology is extended visual inspection, where the direction of sight can be arbitrarily changed to observe the status of the object’s inner surface [64,65]. Additionally there is little damage to the object under inspection [66].

**Figure 17.**
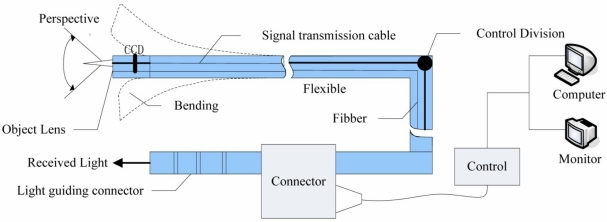
The structure of electronic endoscope.

The basic principle of endoscopic detection technology is the use of visual methods to monitor the inner situation of a sealed object, and then through the optical image evaluation perform testing and diagnosis. The structure of electronic endoscope is shown in Figure 17, with endoscopy, lighting, video processing, video display and image recording. The biggest feature is the use of CCD components to observe objects and convert the optical signals into digital signals, and then these signals are transmitted to the video processing equipment for further processing, display and data extraction. The electronic endoscope is very important for detection of internal damage in engines and other machinery for fault monitoring and diagnosis [67].

Endoscopic detection was first used for medical examination; in the 1950s, with the emergence of specialized industrial endoscopes, this technology was applied to industrial inspection. In the 1960s, endoscopic inspection was used in the engine maintenance field [68]. Currently, endoscopic testing is important for NDT and fault inspection and monitoring of aircraft engines [69].

Endoscopy technology has been developed for over 100 years. It can be divided into three stages, the rod-lens hard endoscope; flexible optical-fibre endoscopy; and flexible electron endoscopy. Significant improvements have been made recently with the rapid development of various fields of science and engineering, influencing endoscopic imaging systems in experimental and industry use. Among these are technologies that improve the endoscope itself in terms of providing new visual features, combining with computer technology, using new illumination techniques. Other developments concern the improvement of image resolution, reduction of scope size and color fidelity using new charge-coupled device (CCD) sensors or alternative techniques for image creation [70]

In the early 1990s, the US Optical Hybrides company produced the Harry McKinley’s “McKinley patent apparatus” and took three years to develop a three-dimensional endoscopic optical system based on a real-time surgical endoscopic imaging system [71]. The US AST Company purchased this technology to create an abdominal cavity three-dimensional display system, the introduction of this system caused widespread interest in endoscopic technology field. In 1998, the Olympus Corporation introduced measureable endoscopes, IV6C6 endoscopic series and IV8C6 series measurement system [72,73]. These systems make full use the principle of the triangulation of stereo vision, by employing dual CCD to mimic the human vision system. Meanwhile, these systems also used other Japanese advanced technologies in the micro-mechanical, optical manufacturing, electronics manufacturing, electronic transmission; they made the smallest diameter of inside glimpse down to 6mm; the direction of the probe can be easily manipulated by using handle manipulation, the probe could bending up to 120°.

Combination with state-of-the-art computer technologies enables modern endoscopic technologies with better quality and new features. There are three main developing directions: automatic, 3D, and in-situ endoscopic NDT. Automation of endoscope can be classified into two aspects: remote control through wireless image transmission, and wired probes capable of auto-tracking. Latest 3D endoscopic display technology is the Real-Depth technology developed in the US, which does not require any additional display equipment. In-situ endoscopic NDT can save much time and cost especially in cases like real-time aircraft diagnostics, so has very high application value.

### 2.5. Terahertz (THz) Technology

THz waves refer to the electromagnetic waves with frequency ranging from 0.1 THz to 10 THz. THz wavelength ranges approximately 0.03 mm to 3 mm, in between microwave and infrared. In THz NDT, usually people use THz radiation with known wavelength to illuminate the object, and receive the THz wave at or near the radiation source after the interaction of the object. The internal structure of the object is determined by analyzing changes of the THz signal, making use of the impact to the THz signal from the object dielectric characteristics or the discontinuity within the object. Since the mid-1980s, terahertz (THz) radiation research has made important progress. Because THz waves have better penetration on most of dry, non-metallic material (such as foam, ceramics, glass, resin, paint, rubber and composite materials, *etc*.) [74] it is applied in NDT. It can be divided into continuous THz and pulse THz technologies. Compared with other NDT techniques, THz imaging has unique advantages in the detection of internal defects for non-metallic material. The THz wave can pass through opaque materials (such as fabrics and plastics) and detect internal defects which visible light cannot detect. It can also be used for insulating materials, unlike IR theromography.

**Figure 18.**
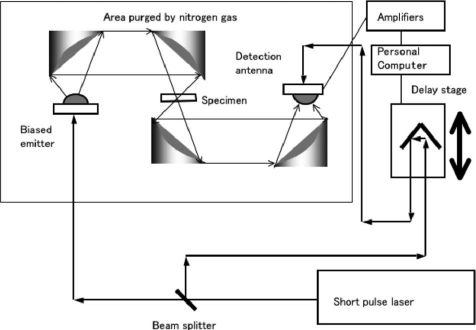
A standard THz-TDS imaging system. Reproduced with permission from [75], copyright 2007, IEEE.

**Figure 19.**
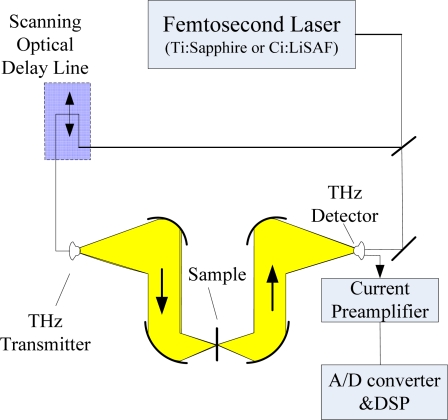
T-ray imager schematic. Reproduced with permission from [76], copyright 2002, IEEE.

There are two kinds of THz Imaging: Passive THz Imaging and Active THz Imaging. Figure 18 shows an example of a standard THz-TDS system with PC antennas [75]. There is a silicon semispherical lens attached to the biased emitter and detection antenna to reduce the reflection of terahertz waves by the boundary of the emitter substrate and air. There is an insensitive region in measuring terahertz waves, because of the reststrahlen band, which is typically 5–10 THz. As it can be seen from Figure 19, an active scanning THz imaging system is a very simple extension of a standard THz-TDS system. It consists of a femtosecond laser, a computer-controlled optical delay line, an optically gated terahertz transmitter, a set of off-axis paraboloidal mirrors for collimating and focusing the terahertz beam, the sample to be imaged, an optically-gated terahertz receiver, a current preamplifier, and a digital signal processor controlled by a personal computer [76].

The THz imaging technique is based on the use of THz electromagnetic waves to detect and identify concealed explosives, chemical and biological agents and illegal drugs through their characteristic transmission or reflectivity spectra in the THz range. Compared to optical images, THz imaging can also provide the information of the concealed objects. Terahertz waves penetrate most dry, nonmetallic and nonpolar objects like plastics, paper, cardboard and nonpolar organic substances. It does not constitute a human radiation hazard (unlike microwaves) and provides better contrast for soft materials [76].

**Figure 20.**
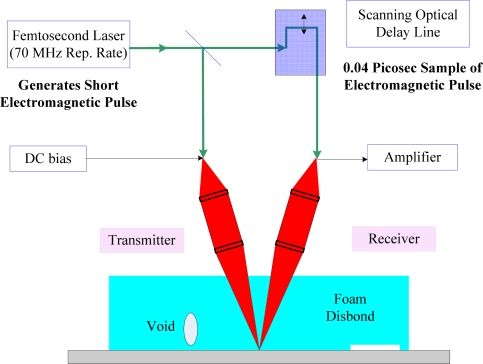
A reflected pulse THz systems used for detection of SOFI [78].

The most important application for THz technology is in the area of THz time-domain spectroscopy or T-ray imaging [76,77]. A typical case of successful application of THz wave technology is the defect detection in space shuttle foam isolation SOFI layers. Indeed, studies have shown that THz waves can provide effective NDT for the shuttle fuel tank insulation materials. THz wave imaging was selected by NASA as one of the four technologies used to detect defects for future launches (the other three are ultrasound, heat waves and infrared rays). Figure 20 shows a reflected pulse THz system used for detection of SOFI [78].

During this period, the detection system and signal processing associated with THz detection technology have developed greatly. A portable continuous-wave THz imaging system is designed and built by Zhang *et al.* which has been approved by NASA for the space shuttle foam insulation testing [79]. Picometrix Company has developed a THz imaging system for large areas and employed the high-speed time domain for non-destructive evaluation of bubbles [79]. There are also many studies of THz NDT data processing, Chiou *et al.* proposed a direct approach for detection and extraction of defects responses to overcome the traditional indirect method (using the reflection from the metal base) limitations [80–82].

In the SOFI THz imaging field, Nair *et al.* used wavelet-based signal processing techniques for image enhancement [83]. Winfreew *et al.* obtained a significant increase in the signal contrast changes between the defects and non-uniformity caused by the foam through signal processing (Morlet wavelet transform, derivative of the frequency domain data, *etc*.) [78]. Roth has presented a variety of signal processing methods used to improve image resolution of SOFI THz method. The results show that the centroid of reflectivity power spectral density has advantages than traditional imaging parameters [84]. In order to accurately compare these data processing methods (such as time-domain signal processing, fast Fourier transform, power spectral density, frequency derivative and the main peak before the measured defect reflection signal), Aldrin provided a quantitative assessment protocol for the SOFI THz detection signal processing methods [85].

In recent years, there are many new applications of THz for NDT, especially in the aerospace field. In addition to the space shuttle external tank foam detection, THz technology is also used for a number of other key materials and structural testing, THZ was used to successfully detect defects (such as debonding) in the tile adhesive layer [86] in the thermal protection material of the Orbiter spacecraft.

The US Army Research Laboratory and NASA have studied the capacity of THz pulse imaging for corrosion detection under paint. Experimental results show that, the thickness of paint and corrosion make the nominal smooth surface become rough and irregular, and the THz waves may be used for corrosion detection [87]. THz technology has also been used for carbon composite materials parts in aircraft structures (carbon fibre is conductive, the detection of such materials are usually slightly beyond the range of THz detection topics), studies have shown that THz imaging can provide quantitative information about the scope of thermal damage and severity of injury [88]. THz imaging has been used to detect defects in the aircraft stringer which is made of polyurethane and epoxy resin foam [89].

Cunlin Zhang developed THz NDT hardware and image processing technology for aerospace use [90–92]. They have successfully made China’s first 0.2 THz continuous wave imaging system, and also implemented pulse THz imaging.

Other applications of THz NDT are as follows: employing THz technology for the thermal stress damage detection of the ceramic ball bearings, which is a time-domain technique that can be used to determine the existence of cracks in ceramic materials [93]; use of THz radiation to detect micro flaws in soft sealed plastic packaging, which relies on the facts that absorption coefficient between plastic and water is very different and refractive index between the plastic and the air is different so that THz technology can detect the closed area and inflatable water-filled defects [94]; using THz time domain spectroscopy imaging for the breaking of glass fibre reinforced epoxy tensile specimens, which could detect the micro-cracks caused by the tensile load and lamination defects [95]; inspection of steel and other metal surfaces: if the surface lateral dimensions are greater than or the same magnitude as the wavelength of THz pulse, THz imaging can be used to identify small surface defects (such as protrusions, scratches, *etc*.) [96].

Pulse THz imaging has been used in plastic welding joint inspection, contaminations in two polyethylene plates welded joints and defects within the pollutants can be detected [97]. THz time-domain technology for ground radar antenna cover plate inspection, composed of advanced composite materials (dielectric material is generally sandwich-type structure, such as glass fibre reinforced resin surface layer and foam core). THz imaging demonstrated stratification for locating water seepage and identification capability [98].

Although the study of THz NDT is not as longer as other NDT technologies, it has high application potential. Development of THz NDT will focus on study of more practical THz source and key hardware equipments, further clarification of the interaction mechanism between THz pulses and the object, modeling and analysis technology of THz testing, and realization of complete quantitative analysis of defects.

## 3. Discussion and Conclusions

This article outlines the various optical sensing and detection methods applied in NDT. This investigation attempted to establish the probability of detecting barely visible damage with optical NDT methods such as digital shearography, ESPI and infrared thermography. Compared to traditional NDT methods, these optical NDT techniques provide a wide range of advantages such as resistance to electromagnetic interference, non-contact, whole-field (except for ESPSI, which is a hole-field technique), not limited to particular material types, real time results (depending on some certain material types) and have been proven reliable in a large number of applications in the laboratory/factory/field environment [99]. In addition, optical NDT methods provide higher sensitivity and resolution for high precision applications. Despite the high cost of optical NDT methods, the advantages over conventional NDT methods mean its expansion into various applications.

As laser-based modalities optical NDT methods have poor penetration, recent development of pulsed eddy current thermography can overcome this limitation [100]. The normal laser-based speckle interferometery optical technique is good at observing surface defects. Laser-ultrasound detection system has been used for remote characterization of subsurface flaws. A pulsed Nd—YAG laser system was used to generate the ultrasonic source whilst a He-Ne laser interferometer detected the subsequent surface displacements. Currently, optical NDT techniques are widely used in a variety of areas such as aerospace, civil, medical, petrochemical, which is leading to further optical NDT technology research including the development of integrated electromagnetic NDT imaging systems.

## References

[1] Xiao NH (2004). New Technologies and Technical Standards for Modern Non-Destructive Testing Technology and Application.

[2] McCann DM, Forde MC (2001). Review of NDT methods in the assessment of concrete and masonry structures. NDT&E Int.

[3] Shen GT (2006). Review of non-destructive testing in China. Insight.

[4] Ansari F (1997). State-of-the-art in the applications of fibre-optic sensors to cementitious composites. Cem. Concr. Compos.

[5] Lin YB, Lai JS, Chang KC, Li LS (2006). Flood scour monitoring system using fibre bragg grating sensors. Smart Mater. Struct.

[6] Tennyson RC, Mufti AA, Rizkalla S, Tadros G, Benmokrane B (2001). Structural health monitoring of innovative bridges in canada with fibre optic sensors. Smart Mater. Struct.

[7] Lin YB, Pan CL, Kuo YH, Chang KC (2005). Online Monitoring of highway bridge construction using fibre bragg grating sensors. Smart Mater. Struct.

[8] Huston DR, Fuhr PL, Beliveau JG Bridge Monitoring with Fibre Optic Sensors.

[9] Chan THT, Yu L, Tam HY, Ni YQ, Liu SY, Chung WH, Cheng LK (2006). Fiber Bragg grating sensors for structural health monitoring of Tsing Ma bridge: Background and experimental observation. Eng. Struct.

[10] Tay AK, Wilson DA, Wood RL (1990). Microdamage and optical signal analysis of impact induced fracture in smart structures. Proc. SPIE.

[11] Murukeshan VM, Chan PY, Ong LS, Asundi A (2000). Effects of different parameters on the performance of a fiber polarimetric sensor for smart structure applications. Sens. Actuat. A Phys.

[12] Thursby G, Sorazu B, Dong F, Culshaw B (2003). Damage detection in structural materials using polarimetric fibre optic sensors. Proc. SPIE.

[13] Steinchen W, Yang LX (2003). Digital Shearography—Theory and Application of Digital Speckle Pattern Interferometry.

[14] Hung YY, Hovanesian JD Nondestructive Evaluation of Composite Material Structures by Shearography.

[15] Yang LX, Siebert T, Caulfield HJ, Vikram C (2008). Digital Speckle Interferometry in Engineering. New Directions in Holography and Speckle.

[16] Shang H, Gao J (2009). Theories and industrial applications of optical interferometric NDT techniques: A review. Insight.

[17] Hung YY, Shang HM, Yang LX (2003). Unified approach for holography and shearography in surface deformation measurement and nondestructive testing. Opt. Eng.

[18] Francis D, Tatam RP, Groves RM (2010). Shearography technology and applications: A review. Meas. Sci. Technol.

[19] Gülker G, Hinsch KD, Hölscher C, Meinlschmidt P, Wolff K (1993). Mapping of plaster detachments in historical murals by electronic speckle pattern interferometry (ESPI). Proc. SPIE.

[20] Groves RM, Pradarutti B, Kouloumpi E, Osten W, Notni G (2009). 2D and 3D non-destructive evaluation of a wooden panel painting using shearography and terahertz imaging. NDT&E Int.

[21] Yang LX, Wegner R, Ettemeyer A Strain and Stress Analysis by Means of a Novel Sensor: MicroStar (Q-100).

[22] Rao MV, Samuel R, Ananthan A (2003). Applications of electronic speckle interferometry (ESI) techniques for spacecraft structural components. Opt. Lasers Eng.

[23] Raman RKS, Bayles R (2006). Detection of decohesion/failure of paint/coating using electronic speckle pattern interferometry. Eng. Fail. Anal.

[24] Lu P (2006). Speckle interference pattern technology and its application in three-dimensional deformation field for diesel pump model measurement. Diesel Eng.

[25] Li XD, Peng Y (2006). Investigation of capillary adhesion between the microcantilever and the substrate with electronic speckle pattern interferometry. Appl. Phys. Lett.

[26] Huang YH, Ng SP, Liu L, Li CL, Chen YS, Hung YY (2009). NDT&E using shearography with impulsive thermal stressing and clustering phase extraction. Opt. Lasers Eng.

[27] Sutton MA, Wolters WJ, Peters WH, Ranson WF, McNeill SR (1983). Determination of displacements using an improved digital correlation method. Image Vis. Comput.

[28] Chu TC, Ranson WF, Sutton MA, Peters WH (1985). Applications of digital image correlation techniques to experimental mechanics. Exp. Mech.

[29] Sutton MA, Cheng MQ, Peters WH, Chao YJ, McNeill SR (1986). Application of an optimized digital correlation method to planar deformation analysis. Image Vis. Comput.

[30] Sutton MA, McNeill SR, Jang J, Babai MK (1988). Effects of subpixel image restoration on digital correlation error estimates. Opt. Eng.

[31] Peters WH, Sutton MA, Poplin WP, Walker DM (1989). Whole field experimental displacement analysis of composite cylinders. Exp. Mech.

[32] Sutton MA, Turner JL, Bruck HA, Chao TL (1992). Experimental investigations of three-dimensional effects near a crack tip using computer vision. Int. J. Fract. Mech.

[33] Luo PF, Chao YJ, Sutton MA (1993). Accurate measurement of three-dimensional deformations in deformable and rigid bodies using computer vision. Exp. Mech.

[34] Sutton MA, McNeill SR, Helm JD, Chao YJ, Rastogi PK (2000). Advances in two-dimensional and three-dimensional computer vision. Topics in Applied Physics, Photomechanics.

[35] Han G, Sutton MA, Chao YJ (1994). A study of stationary crack-tip deformation fields in thin sheets by computer vision. Exp. Mech.

[36] Sutton MA, Deng X, Liu J (1996). Determination of elastic-plastic stresses and strains from measured surface strain data. Exp. Mech.

[37] Lu H Statistical Analysis of the Random Error in Measurements Obtained Using Digital Correlation of Speckle Patterns.

[38] Anwander M, Hadrboletz A, Weiss B (1999). Thermal and mechanical properties of micromaterials using laser optical strain sensors. Proc. SPIE.

[39] Wattrisse B, Chrysochoos A, Muracciole JM, Nemoz-Gaillard M (2000). Analysis of strain localisation during tensile test by digital image correlation. J. Exp. Mech.

[40] Zhou P, Goodson KE (2001). Subpixel displacement and deformation gradient measurement using digital image/speckle correlation (DISC). Opt. Eng.

[41] Roux S (2006). Stress intensity factor measurements from digital image correlation: Post-processing and integrated approaches. Int. J. Fract.

[42] Shi XQ (2004). In-Situ micro-digital image speckle correlation technique for characterization of materials properties and verification of numerical models. IEEE Trans. Compon. Packag. Technol.

[43] Coburn D, Slevin J (1995). Digital correlation system for nondestructive testing of thermally stressed ceramics. Appl. Opt.

[44] Kirugulige MS (2007). Measurement of transient deformations using digital image correlation method and high-speed photography: Application to dynamic fracture. Appl. Opt.

[45] Roux S, Rethore J, Hild F (2009). Digital image correlation and fracture: An advanced technique for estimating stress intensity factors of 2D and 3D cracks. J. Phys. D: Appl. Phys.

[46] Cirello A, Pasta S (2010). Displacement measurement through digital image correlation and digital speckle pattern interferometry techniques in cold-expanded holes. Strain.

[47] Chan YC, Yeung F, Jin G (1995). Nondestructive detection of defects in miniaturized multilayer ceramic capacitors using digital speckle correlation techniques. IEEE Trans. Compon. Packag. Manuf. Technol.

[48] Lyons JS, Liu J, Sutton MA (1996). High-temperature deformation measurements using digital-image correlation. Exp. Mech.

[49] Reu PL, Miller TJ (2008). The application of high-speed digital image correlation. J. Strain Anal. Eng. Des.

[50] Zhang YP, Zhu HN, Zhou WL, Liu HF (2002). Application of the fourier transform in electronic speckle photography. Exp. Mech.

[51] Yang Y, Wang Y, Li M (2006). Research of high–accuracy digital image correlation measurement system. Acta Opt. Sin.

[52] Chen D, Gu J, Jiang J (2005). Study on the digital speckle correlation method for in-plane displacement measurement in the case of slant optical axis. Acta Opt. Sin.

[53] Xavier M (1992). Infrared Methodology and Technology [M]. Nondestructive Testing Monographsand Tracts.

[54] Roderic KS, Patric OM, Paul MI (1995). Special Nondestructive Testing Methods [M]. Nondestructive Testing Handbook.

[55] Quinn TJ, Compton JP (1975). The foundations of thermometry. Rep. Prog. Phys.

[56] Lee RD, Kostkowski HJ, Quinn TJ (1973). Temperature: Its Measurement and Control in Science And Industry.

[57] Meola C, Carlomagno GM (2006). Application of infrared thermography to adhesion science. J. Adhes. Sci. Technol.

[58] Sfarra S, Ibarra-Castanedo C, Avdelidis NP (2010). A comparative investigation for the non-destructive testing of honeycomb structures by holographic interferometry and infrared thermography. J. Phys.

[59] Rantala J, Wu D, Busse G (1998). NDT of polymer materials using lock-in thermography with water-coupled ultrasonic excitation. NDT&E Int.

[60] Maierhofer C, Arndt R, Rollig M, Rieck C (2006). Application of impulse-thermography for non-destructive assessment of concrete structures. Cem. Concr. Compos.

[61] Inagaki T, Ishii T, Iwamoto T (1999). On the NDT and E for the diagnosis of defects using infrared thermography. NDT E Int.

[62] Datdma V, Marcuccio R, Pappalettere C, Smith GM (2001). Thermographic investigation of sandwich structure made of composite material. NDT E Int.

[63] Wilson J, Tian GY, Mukriz I, Almond D (2011). PEC thermography for imaging multiple cracks from rolling contact fatigue. NDT & E Int.

[64] Zhang Y (2004). The importance of borescope detection work in the engine maintenance. Aviat. Maint. Eng.

[65] Li CY, Shi H, Yao HY (2006). Characteristics of the borescope imaging. Aviat. Maint. Eng.

[66] Samsonov P (1993). Remote visual inspection for NDE in power plants. Mater. Eval.

[67] Yu H (2005). Borescope and its application in aero engine maintenance. Aeronaut. Manuf. Technol.

[68] Hirose S, Ikuta K, Tsukamoto M (1990). Development of a shape memory alloy actuator (Measurement of material characteristics and development of active endoscopes). Adv. Robot.

[69] Anon (1998). Video Borescoping helps keep gas turbines healthy. Turbomach. Int.

[70] Schurr MO, Kunert W, Arezzo A (1999). The role and future of endoscopic imaging systems. Endoscopy.

[71] Kaplan H (1994). A borescope that behaves like the human eye. Photon. Spectra.

[72] Wu S, Zhao JH, Huang ZJ (2000). The application of measureable video endoscope in non-destructive testing. Aerosp. Mater. Technol.

[73] Ross I Anglo Japanese Collaboration in the Design of Medical and Industrial Endoscopic Equipment.

[74] Mittleman DM, Jacobsen RH, Nussm C (1996). T-ray imaging. IEEE J. Sel. Top. Quantum Electron.

[75] Hosako I, Sekine N, Patrashin M, Saito S, Fukunaga K, Kasai Y, Baron P, Seta T, Mendrok J, Ochiai S, Yasuda H (2007). At the dawn of a new era in terahertz technology. Proc. IEEE.

[76] Siegel PH (2002). Terahertz technology. IEEE Trans. Microw. Theory Tech.

[77] Arnone DD (1999). Applications of terahertz (THz) technology to medical imaging. Proc. SPIE.

[78] Winfreew P, Madaras EI Detection and Characterization of Flaws in Sprayed on Foam Insulation with Pulsed Terahertz Frequency Electromagnetic Waves.

[79] Karpowicz N, Zhong H, Xu J, Lin K-I, Hwang J-S, Zhang X-C (2005). Non-destructive Sub-THz CW Imaging. Proc. SPIE.

[80] Zimdars D, White JS, Stuk G (2006). Large area terahertz imaging and non-destructive evaluation applications. Insight.

[81] Chiou CP, Thompson RB, Winfreew WP, Madaras EI, Seebo J (2006). Modeling and Processing of Terahertz Imaging in Space Shuttle External Tank Foam Inspection. Quant. Nondestruct. Eval.

[82] Chiou CP, Thompson RB, Winfr WP, Madaras EI, Seebo J (2007). Processing Terahertz ray data in space shuttle inspection. Quant. Nondestruct. Eval.

[83] Nair NV, Melapudiv VR, Vemulapalli P, Ramakrishnan S, Udpa L, Udpa SS, Winfree WP (2006). A Wavelet based signal processing technique for image enhancement in Terahertz imaging data. Quant. Nondestruct. Eval.

[84] Roth DJ, Seebo JP, Walker JL, Aldrin JC (2007). Signal processing approaches for Terahertz data obtained from inspection of the shuttle external tank thermal protection system foam. Rev. Prog. Quant. Nondestruct. Eval.

[85] Aldrin JC, Roth DJ, Seebo JP, Winfree WP (2007). Protocol and assessment of signal processing and feature extraction methods for Terahertz NDE for spray-on foam insulation. Rev. Prog. Quant. Nondestruct. Eval.

[86] Karpowicz N, Zhong H, Zhang C (2005). Compact continuous-wave sub-terahertz system for inspection applications. Appl. Phys. Lett.

[87] Anastasir F, Madaras EI (2005). Terahertz NDE for under paint corrosion detection and evaluation. Quant. Nondestruct. Eval.

[88] Redo-Sanchez A, Karpowicz N, Xu J, Zhang X-C Damage and Defect Inspection with Terahertz Waves.

[89] Bechmann J, Richter H, Zscherpel U, Ewert U, Weinzierl J, Schmidt L-P, Hochfrequenztechni LF, Erlangen U, Rutz F, Koch M, Richter H, Hübers H-W Imaging Capability of Terahertz and Millimetre-Wave Instrumentations for NDT of Polymer Materials.

[90] Zhao G, Sun H, Tian Y (2006). Optical system for application of THz spectroscopy and THz imaging. Proc. SPIE.

[91] Zhang ZW (2006). Study of Pulsed THz Time-Domain Spectroscopic Imaging and THz Continuouswave Imaging.

[92] Zhang Z, Cui W, Zhao G (2006). Data processing methods for terahertz transmitted spectral imaging. Proc. SPIE.

[93] Reitenm T, Hess L, Cheville RA (2006). Nondestructive evaluation of ceramic materials using terahertz impulse ranging. Proc. SPIE.

[94] Morita Y, Dobroiu A, Kawase K (2005). Terahertz technique for detection of micro leaks in the seal of flexible plastic packages. Opt. Eng.

[95] Rutz F, Wietzke S, Koch M Non-Destructive Testing of Glass-Fibre Reinforced Polymers Using Terahertz Spectroscopy. http://www.ndt.net/article/ecndt2006/doc/We.2.8.2.pdf.

[96] Loffler T, Hils B, Roskosh G Characterization of Surface Structures Using THz Radar Techniques with Spatial Beam Filtering and out-of-focus Detection. http://www.ndt.net/article/ecndt2006/doc/Tu.2.8.3.pdf/.

[97] Wietzke S, Jordens C, Krumbholz N (2007). Terahertz imaging: A new non-destructive technique for the quality control of plastic weld joints. J. Eur. Op. Soc. Rapid Publ.

[98] Zimdars D, White J, Sucha G (2007). Terahertz measurement and imaging detection of delamination and water intrusion in ground based radome panels. Proc. SPIE.

[99] Gryzagoridis J, Findeis D (2010). Impact damage detection on composites using optical NDT techniques. Insight.

[100] Al-Qubaa AR, Tian GY, Wilson J, Woo WL, Dlay SS (2010). Feature extraction using normalized cross-correlation for pulsed eddy current thermographic images. Meas. Sci. Technol.

